# Multilingual competencies among ambulatory care providers in three German Federal States

**DOI:** 10.1186/s12875-022-01926-1

**Published:** 2022-12-06

**Authors:** Frank Müller, Harland Holman, Eva Hummers, Dominik Schröder, Eva Maria Noack

**Affiliations:** 1 Department of General Practice, University Medical Center Göttingen, Humboldtallee 38, 37073 Göttingen, Germany; 2 Department of Family Medicine, College of Human Medicine, Michigan State University, 15 Michigan St NE, Grand Rapids, MI 49503 USA

**Keywords:** Language proficiency, Primary care, Migration, Language barrier

## Abstract

**Background:**

Providing medical care to newly arrived migrants presents multiple challenges. A major challenge is a lack of a common language in the absence of language interpretation services. We examine the multilingualism of German physicians and clinical psychotherapists providing ambulatory care.

**Methods:**

We retrieved publicly available data from the Associations of Statutory Health Insurance Physicians provider registry of three German federal states (Lower Saxony, Saarland, Bavaria). We selected and grouped relevant practice-based disciplines. We used descriptive statistics to analyze the provider’s multilingualism among different disciplines.

**Results:**

69.6% of ambulatory providers offer consultations only in German. 15.5% of providers reported offering consultations in one additional non-German language, and 14.9% in two or more additional languages. Most common additional languages were English (28.6%) and French (9.9%). 1.4% of providers reported offering consultation in at least one language of the Middle Eastern region (Arabic, Dari, Hebrew, Kurdish, Pashtu, Farsi, and Turkish). There were differences in the offered languages between the medical disciplines with the highest mean rates found for gynecologists and obstetricians, urologists, and general surgeons. Psychotherapeutic disciplines offered consultation in other languages significantly less often.

**Conclusion:**

Our study suggests a significant numeric mismatch in the number of providers offering consultations in the languages of people seeking protection in Germany. The resulting language barriers are compromising equitable access and quality of care.

**Supplementary Information:**

The online version contains supplementary material available at 10.1186/s12875-022-01926-1.

## Background

The number of people fleeing violence, persecution, severe poverty, or increasingly the threats of climate change and migrating to other countries has been constantly increasing in the last decade. The United Nations High Commissioner for Refugees estimates that at mid-2021 84 million people worldwide were forcibly displaced [1]. As a result, immigration is increasingly creating linguistic diversity in many societies of the Western world. The treatment of patients with limited proficiency in the locally spoken language (LPLL) has become more common for many medical care providers [2]. A growing body of evidence demonstrates that the presence of LPLL negatively impacts access to healthcare, receiving and continuing necessary care, and physical health status and outcomes [3–5]. These studies have also revealed that LPLL patients were less likely to obtain satisfactory care, be provided pertinent information, understand treatment plans and disease processes, and trust their physicians [6, 7]. These circumstances make people with LPLL a particularly vulnerable patient group.

Germany admitted over one million refugees from the Middle East in the last decade [8] and is currently one of the main reception countries for Ukrainians fleeing Russian aggression [9]. A representative survey in Germany showed that only 2% of asylum seekers rated their oral German skills as “good” or “very good” at arrival. While many people seeking protection (PSP) attempt mandatory language classes after arrival, this education enables only one to speak German at a basic (B1-)level [10, 11].

Unlike in other countries, such as the United States of America (U.S.A.) [12] or Norway [13], interpreter services are not mandatory nor systematically allotted in German healthcare. As a result, professional interpreters are often not available and providers make use of lay interpreters, such as family members or friends, when attempting to bridge language barriers [2]. One resource in bridging language barriers may be seen in bi- or multilingual practice staff. Studies showed improved outcomes of care when LPLL patients had encounters with language-concordant providers [14–16]. This study aims to assess the availability of multilingual consultation among German ambulatory care providers.

## Methods

Ambulatory care in Germany, including primary and specialized outpatient services, e.g. radiology, is provided by approximately 150,000 physicians and psychotherapists licensed by the Association of Statutory Health Insurance Physicians [17]. Ambulatory providers have approximately 4 million patient contacts each day [18]. By law, the Association of Statutory Health Insurance Physicians in each federal state publishes information about their licensed physicians and psychotherapists [19]. The law requires providers to update this information immediately when changes occur. The register contains the name of the providers and their training, opening hours of practices, contact information, and information on “foreign languages skills” (other than German) that providers offer consultations. The foreign language skills can be the providers’ own language skills or availability of multilingual in-practice nursing staff that can serve as interpreter.

The publicly available data from three German federal states (Lower Saxony [20], Saarland [21], Bavaria [22]) were retrieved from the Association of Statutory Health Insurance Physicians website between 10th March and 19th March 2022. These federal states were selected because they reflect the variety of residential and socioeconomic structures in Germany well and the required data could be extracted without additional efforts. Data included physicians’ entity of specialization, zip code, and indicated foreign languages spoken by the provider or practice staff. To avoid distortions due to uncommon medical disciplines, we have formed groups for practice-based family medicine, internal medicine specialists (including gastroenterologists, pulmonologists, cardiologists, etc.), psychotherapy & counseling (including physicians and clinical psychologists), gynecology & obstetrics, ophthalmology, pediatric counseling & psychiatry (including physicians and psychologists), pediatrics, dermatology, urology, orthopedics & trauma surgery, neurology, otolaryngology, general surgery, and psychiatry. Other practice-based medical disciplines, e.g. radiotherapy, dialysis practices, human genetics, laboratory medicine, occupational health, pathology, or radiology, were excluded from the study because they are often affiliated with hospitals or organized in large practice networks and do not reflect single practice-based care. They might either need additional language proficiencies to provide service (laboratory medicine, pathology) or have access to a hospital or clinic-groups’ resources for language interpreting.

To describe practice locations, we obtained data on population density for each district from the German Federal Statistics Office. Population density was categorized on the district level with 0-299 inhabitants per square kilometer (km²) to be considered rural, 300-1,499 inhabitants/km² semi-dense and more than 1,499 inhabitants/km² an urban agglomeration in accordance with the cut-off values of the Statistical Commission of the United Nations [23].

Additionally we obtained data from German Federal Statistics office on the number of PSPs and their nationality to calculate PSP per multilingual provider for each district [24]. PSPs are defined as all foreigners in Germany that are seeking protection for humanitarian reasons. PSPs include anyone claiming asylum or protection, regardless if protection status has been granted, is pending, or has been rejected [25]. This subanalyses is shown in the appendix (figure S1).

After refining and verifying of the dataset, we used descriptive statistics including raw numbers, percentages, means with corresponding 95% confidence intervals (CI) and standard deviation (SD), to assess the frequency and type of provider that offers consultations in languages other than German. Provider characteristics were compared in regions with different population density using the chi-square test. The PSP per multilingual provider was calculated for each district and plotted. All statistical analyzes were performed using SPSS 27 (IBM, Armonk, NY), figures were plotted using GraphPad Prism 9 (GraphPad Software, San Diego, CA) and choropleth maps were plotted using QGIS Version 3.225 (under GNU License).

## Results

**Fig. 1:**
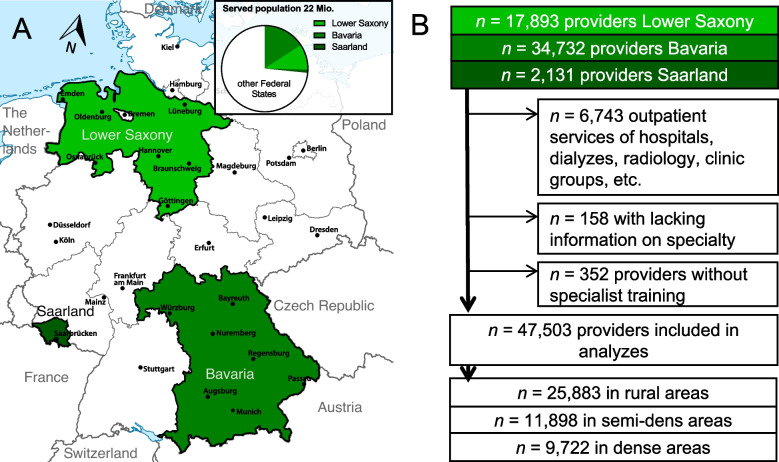
**A **Mapping of included Federal States. **B** Flow chart of inclusion

After applying the exclusion criteria, 47,503 providers were included in the analysis (Fig. 1B). These physicians and psychotherapists provide care to over 22 million residents, slightly more than a quarter of the overall population of Germany (Fig. 1 A). Out of these, 2.7% (*n* = 595,070) were PSPs, mainly migrants from the Middle East (*n* = 347,595).

Most providers (69.6%) did not offer consultations in any language other than German. 15.5% of providers offered consultations in one additional language other than German, while another 14.9% of the providers offered consultations in two or more languages other than German. The additional spoken languages were mainly English (28.6% of all providers), French (9.9% of all providers), Spanish (2.0% of all providers), Russian (2.2% of all providers), and Italian (1.4% of all providers). Consultations in at least one language of the Middle Eastern region (Arabic, Dari, Hebrew, Kurdish, Pashtu, Farsi, and Turkish) were offered by 1.4% of all providers. The providers who offered consultations in foreign languages were significantly more often located in semi-dense districts, while in rural and urban districts multilingual competencies were offered less often (Table 1).

**Table 1:** Characteristics of analyzed providers

	Total	Population density			
		Rural	Semi-dense	Urban	
	n (%)	n (%)	n (%)	n (%)	*p*
Family Medicine	11,358 (23.9)	7,389 (28.5)	2,319 (19.5)	1,650 (17)	<0.001
Internal Medicine	8,585 (18.1)	4,798 (18.5)	2,233 (18.8)	1,554 (16)	<0.001
Psychotherapy & Counseling	7,369 (15.5)	3,115 (12)	1,940 (16.3)	2,314 (23.8)	<0.001
Gynecology & Obstetrics	3,401 (7.2)	1,761 (6.8)	951 (8)	689 (7.1)	<0.001
Ophthalmology	3,014 (6.3)	1,584 (6.1)	750 (6.3)	680 (7)	0.010
Pediatric Counseling & Psychiatry	2,350 (4.9)	1,208 (4.7)	614 (5.2)	528 (5.4)	0.073
Pediatrics	2,104 (4.4)	1,158 (4.5)	582 (4.9)	364 (3.7)	<0.001
Dermatology	1,093 (2.3)	525 (2)	312 (2.6)	256 (2.6)	<0.001
Urology	1,018 (2.1)	565 (2.2)	274 (2.3)	179 (1.8)	0.044
Orthopedics & Trauma surgery	2,983 (6.3)	1,644 (6.4)	782 (6.6)	557 (5.7)	0.018
Neurology	852 (1.8)	386 (1.5)	245 (2.1)	221 (2.3)	<0.001
Otolaryngology	898 (1.9)	393 (1.5)	225 (1.9)	280 (2.9)	<0.001
Surgery	1,161 (2.4)	719 (2.8)	262 (2.2)	180 (1.9)	<0.001
Psychiatry	1,317 (2.8)	638 (2.5)	409 (3.4)	270 (2.8)	<0.001
Total	47,503 (100)	25,883 (100)	11,898 (100)	9,722 (100)	
Additional spoken Languages					
None	33,080 (69.6)	17,413 (67.28)	7191 (60.44)	8,476 (87.18)	<0.001
1	7,340 (15.5)	4,449 (17.19)	2260 (18.99)	631 (6.49)	
2 or more	7,083 (14.9)	4,021 (15.54)	2447 (20.57)	615 (6.33)	
English	13,569 (28.6)	8,022 (30.99)	4373 (36.75)	1,174 (12.08)	<0.001
French	4,705 (9.9)	2,544 (9.83)	1752 (14.73)	409 (4.21)	<0.001
Middle East languages	645 (1.4)	344 (1.33)	258 (2.17)	43 (0.44)	<0.001

**Fig. 2:**
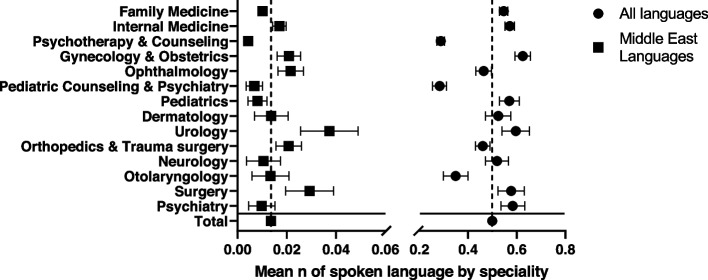
Mean additional spoken languages (with corresponding 95% confidence intervals) among different medical disciplines

The mean number of additional languages among all analyzed providers was 0.49 (min-max 0–8, SD 0.9). The highest mean rates were found among gynecologists and obstetricians, urologists, general surgeons, and psychiatrists, while psychotherapists and counselors for both adults and children as well as otolaryngologists offered significantly less often consultations in languages other than German compared to the overall mean. Consultations in languages spoken in the Middle East regions were only offered by a few providers and considerably more often by Urologists, Orthopedic surgeons and general surgeons (Fig. 2).

The distribution of PSP per multilingual provider on district level is shown in the appendix.

## Discussion

Our study showed that 30.4% of ambulatory care providers offer consultations in languages other than German with English and French as the most common languages.

Multilingual competencies are significantly more common among providers located in districts with semi-dense population compared to urban or rural districts. Consultation of languages that are spoken in the Middle East, the most common region of origin of PSPs in Germany, were only offered by 1.4% of all included providers. Given the large number of PSPs in Germany, our study suggests that multilingual providers do not match the language needs of the LPLL population and is perpetuated by not mandating medical interpreters in ambulatory care.

The number of multilingual psychiatrists and psychotherapists for adults as well as for children and adolescents are particularly low. This shortage is worrying considering the high incidence of mental health problems among refugees [26, 27]. Compared to the number of PSPs in each district, multilingual providers are scarce in rural Bavaria. In these regions, the language competency for Middle Eastern languages is particularly little or even non-existing. This is concerning as PSPs from the Middle East region is by far the largest group of PSPs in Germany.

The lack of language interpretation services has recently been highlighted by the German Medical Association [28]. Many patients bring family members including children to interpret for them in the doctor’s office, which introduces concerns regarding privacy and quality of the translation [2, 29].

Multilingual providers can contribute considerably to the wellbeing of migrant communities: Studies have shown that migrants experience better care when they encounter physicians who can speak to them in their preferred language [15, 16]. Besides advocating for mandatory interpretation services, the integration of migrant physicians and psychotherapists, as well as diversity programs that enable students with migrant or multilingual backgrounds to study or train for a medical profession, could increase the numbers of multilingual providers and eventually lead to better healthcare provision for migrant communities [15, 16]. Research from other Western countries shows that the medical workforce and particularly physician workforce is not very diverse [30], but multilingual physicians tend to practice in areas with high concentration of LPLL people [31].

There are some limitations to consider. First, the information from the providers about their foreign language skills are self-reported. It is therefore not checked how well providers or their practice staff speak the respective language. It is also conceivable that some providers do not report their existing foreign language skills, because they consider them insufficient to hold consultation in this language. This limitation may be negligible as information regarding the providers’ ability to hold consultations in additional languages is a default request for the Association of Statutory Health Insurance Physicians and providers are required by law to keep their information up to date. An additional limitation is the imprecision that no dialectal variants of languages could be considered in this study and languages were grouped together in the analysis. For example, Middle Eastern languages comprises numerous languages and dialectal variants. Also, we only included PSPs in our LPLL group. With this definition, we may not have accounted for LPLL groups such as migrants, foreign students, tourists, seasonal workers etc.

A strength of the study is that data on all ambulatory care providers of three federal states could be included in the study which decreases selection bias.

Besides these potential limitations, the provider register of the Association of Statutory Health Insurance Physicians is the only comprehensive and daily updated information source for the general public about which providers offer consultations in foreign languages. As this register is the only reliable information resource for LPLL persons and their supporters, it virtually defines the offered services.

## Conclusion

Germany has admitted over one million people seeking protection in the last decade of which the overwhelming majority did not speak German at arrival. Still, professional medical language interpretation is not established in ambulatory care. Based on available data, there is a significant numeric mismatch in the number of providers offering consultations in the languages of PSP patients. The resulting language barriers are a major threat to provide high quality and equitable care.

## Supplementary Information

**Additional file 1.** **Figure S1.** People seeking protection per provider that offers consultation in languages other than German. 3B: People seeking protection originating from the Middle East per provider that offers consultation in at least one Middle East language (Arabic, Dari, Hebrew, Kurdish, Pashtu, Farsi, Turkish).

## Data Availability

Analyzed data was retrieved from the Association of Statutory Health Insurance Physicians website (Lower Saxony [20], Saarland [21], Bavaria [22]. Analyzed data tables are available on reasonable request from the corresponding author.
