# Supplementary material for: Multilingual competencies among ambulatory care providers in three German Federal States
Source: BMC Prim Care. 2022 Dec 6;23:315. doi: 10.1186/s12875-022-01926-1 (PMC9724318; doi:10.1186/s12875-022-01926-1)
Supplement: Supplementary file 1 — Additional file 1. Figure S1. People seeking protection per provider that offers consultation in languages other than German. 3B: People seeking protection originating from the Middle East per provider that offers consultation in at least one Middle East language (Arabic, Dari, Hebrew, Kurdish, Pashtu, Farsi, Turkish). [file 12875_2022_1926_MOESM1_ESM.docx]

# Appendix

The number of PSP per provider who offer consultation in at least one foreign language is shown in Figure S1. PSP per provider ranged from 12.1 in the district of Osnabrück in Lower Saxony to 329.3 in the district of Schweinfurt in Bavaria. The mean PSP per provider ratio over all 146 districts was 56.0 (SD 44.4).

If only those PSPs of Middle Eastern origin and providers that offer consultations in at least one Middle Eastern language were considered, the number of PSP per provider was considerably higher (Figure 3B). In 61 of 96 Bavarian and one of 45 districts in Lower Saxony (the district of Wittmund) no provider offered consultation in a Middle Eastern language. The mean PSP from the Middle East per provider offering consultations in a Middle Eastern language was 309.9 (SD 496.6).


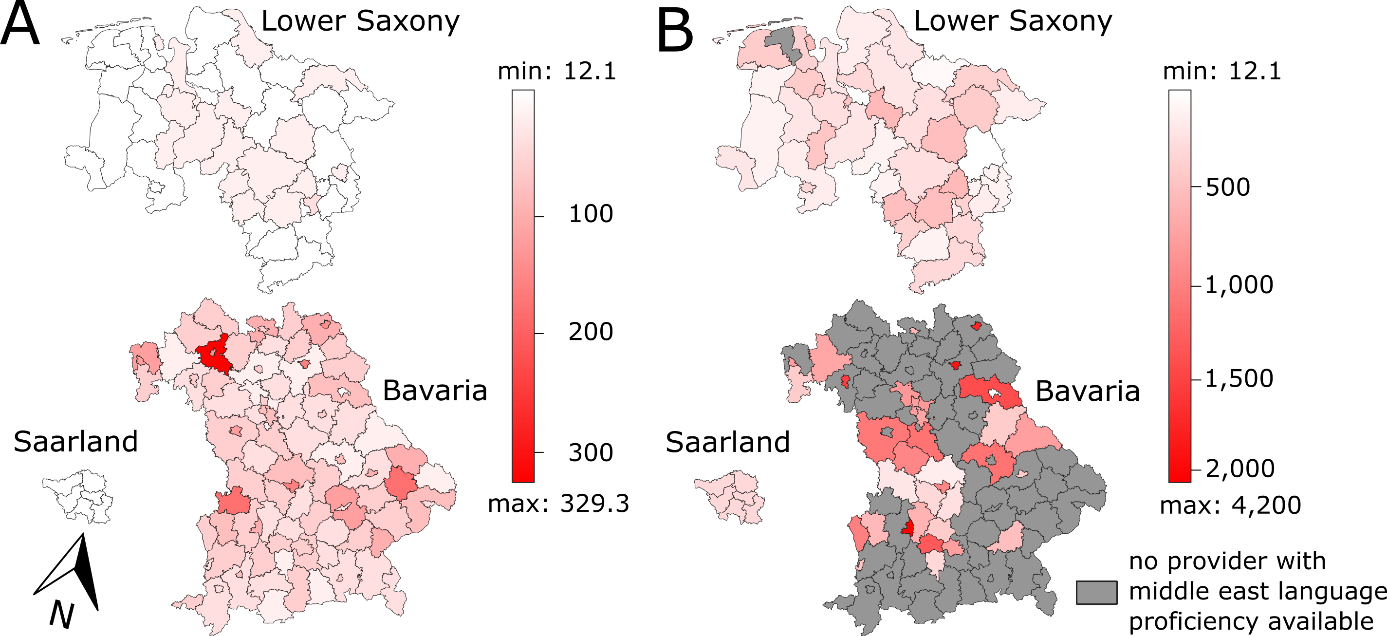


Figure S1: People seeking protection per provider that offers consultation in languages other than German. 3B: People seeking protection originating from the Middle East per provider that offers consultation in at least one Middle East language (Arabic, Dari, Hebrew, Kurdish, Pashtu, Farsi, Turkish).
